# European Society of Neuroendocrine Tumors (ENETS) 2025 guidance paper for lung and thymic carcinoids

**DOI:** 10.1111/jne.70174

**Published:** 2026-04-06

**Authors:** Eric Baudin, Alice Durand, Wieneke Buikhuisen, Jaume Capdevila, Martyn Caplin, Christophe M. Deroose, Clarisse Dromain, Antongiulio Faggiano, Pier Luigi Filosso, Gregory Kaltsas, Marco Volante, Thomas Walter, Rocio Garcia‐Carbonero

**Affiliations:** ^1^ Department of Endocrine Oncology and Nuclear Medicine Gustave Roussy Cancer Institute, Paris‐Saclay University Paris France; ^2^ Department of Medical Oncology Hospices Civils de Lyon, Hôpital Édouard Herriot, Université de Lyon Lyon France; ^3^ Department of Thoracic Oncology Netherlands Cancer Institute Amsterdam The Netherlands; ^4^ Department of Medical Oncology Vall d'Hebron University Hospital, Vall Hebron Institute of Oncology (VHIO) Barcelona Spain; ^5^ Department of Gastroenterology, Neuroendocrine Tumour Unit, ENETS Centre of Excellence, Royal Free Hospital London UK; ^6^ Nuclear Medicine Unit University Hospitals Leuven Leuven Belgium; ^7^ Nuclear Medicine and Molecular Imaging, Department of Imaging and Pathology KU Leuven Leuven Belgium; ^8^ Department of Diagnostic and Interventional Radiology Lausanne University Hospital and University of Lausanne Lausanne Switzerland; ^9^ Endocrinology Unit, Department of Clinical and Molecular Medicine Sant'Andrea Hospital, ENETS Centre of Excellence, Sapienza University of Rome Rome Italy; ^10^ Department of Thoracic Surgery San Giovanni Battista Hospital, University of Torino Turin Italy; ^11^ Department of Propaedeutic and Internal Medicine National and Kapodistrian University of Athens Athens Greece; ^12^ Department of Oncology University of Turin at San Luigi Hospital Orbassano Torino Italy; ^13^ Department of Medical Oncology Hospital Universitario 12 de Octubre, Imas12 Research Institute, Medicine Faculty, Universidad Complutense de Madrid Madrid Spain

**Keywords:** guidance, lung carcinoid, neuroendocrine, thymic carcinoid, treatment

## Abstract

This ENETS guidance paper, developed by a multidisciplinary working group, provides up‐to‐date and practical advice on the diagnosis and management of lung and thymic carcinoids, based on recent developments and study results. These recommendations aim to provide practical recommendations for the diagnosis, treatment and follow‐up of these tumours, and pave the road for more standardised care for our patients expecting improved outcomes. This paper is structured on a question‐answer format in order to address common dilemmas encountered in clinical practice, including controversial issues and areas of uncertainty, based on the best available evidence and expert opinion when good quality evidence is not available. Each recommendation will provide a level of evidence and grade of recommendation as per the GRADE system (adapted from the Infectious Disease Society of United States Public Health Service grading system).

## 1 INTRODUCTION

ENETS has initiated several collaborative interactions between specialists from multiple disciplines dealing with the diagnosis and treatment of neuroendocrine neoplasms (NENs), with the double goal of checking progress on the most critical questions that affect NENs management and providing practical guidance to caregivers on clinical situations they may encounter in ‘real life’. This guidance policy acknowledges the fact that a high level of evidence is generally lacking for many clinical scenarios, and physicians treat patients with NENs based on the best available evidence or experience of experts from large referral centres, while keeping the ambition to support the development of future studies to reach the highest level of evidence on every question. This approach reflects the rarity and complexity of this family of neoplasms.

This guidance manuscript deals with the diagnosis and therapeutic management of lung and thymic neuroendocrine tumours (NETs), and is organised in ‘a question and answer’ manner. Most relevant questions have been selected by the ENETS Advisory Board (Table S1). Answers are structured in a similar way including a summary of current knowledge, consensus on the best answer to the question, and specific considerations regarding thymic carcinoids or Multiple Endocrine Neoplasia type 1 (MEN1) status. Grade and level of evidence are provided for each recommendation (Table S2), although expert multidisciplinary team decisions in centres of excellence are nevertheless highly advised for all patients.

## 2 CHARACTERISATION PRIOR TO THERAPY

### 2.1 Histology

Q1: Is there an added value of Ki‐67 or other pathological or molecular markers to the current WHO 2021 classification of lung and thymic carcinoids?

The 2021 *World Health Organization (WHO)* classification of *Thoracic Tumours* stratifies lung (LC) and thymic (ThC) well differentiated NETs into two main prognostic subtypes, typical (TC) and atypical (AC) carcinoids, based on morphological differentiation, mitotic count and the presence or not of necrosis on primary tumour specimens (Table 1).^1^ The term carcinoid/NET not otherwise specified (NOS) is indicated in non‐surgical samples as artefacts or limited tumour tissue sampling may preclude adequate classification in biopsies/cytological samples in primary and metastatic lesions. Histopathological classification is influenced by inter‐observer variability and may benefit from novel approaches such as digital pathology and artificial intelligence tools in the near future.^2^, ^3^

**TABLE 1:** WHO 2021 Classification of lung and thymic carcinoids.

	Classification	Diagnostic criteria	Ki67 index
Well differentiated neuroendocrine tumor (NET)	Typical carcinoid	< 2 mitosis/2 mm^2^ and no necrosis	Up to 5%
	Atypical carcinoid	2–10 mitosis/2 mm^2^ and/or punctate necrosis	Up to 20%
	Carcinoids/NETs with elevated mitotic counts and/or Ki67 proliferation index^a^	Atypical carcinoid morphology and a higher proliferative index (>10 mitosis/2 mm^2^)^a^	>20%
Poorly differentiated neuroendocrine carcinoma (NEC)	Large cell NEC	>10 mitosis/2 mm^2^, virtually always necrosis and large cell cytomorphology	40%–80%
	Small cell NEC or small cell (lung) carcinoma (SCLC)	>10 mitosis/2 mm^2^, virtually always necrosis and small cell cytomorphology	50%–100%

^a^ Proposed (in the text of the WHO 2021 classification) as equivalent to G3 NET of the WHO classification for digestive tumors, but not a formally recognised category.

In 2022, a common terminology of NEN (including NET and NEC) across primaries was proposed by the *International Agency for Research on Cancer* (IARC) and WHO, adopting for lung and thymus TC and AC the terms NET grade (G)1 and G2, respectively, although Ki‐67 is not required for the LC and ThC WHO histopathological classification.^4^ Indeed, there is currently no robust demonstration that Ki‐67 index determination or any other markers have any major added prognostic value in the group of carcinoids/NETs as compared to the current WHO classification.^5^, ^6^ Limitations for such demonstration include the low number of events (recurrence or death) due to the excellent prognosis of TC and the low proportion of patients with AC, mixed LC populations, but also to the absence of large multicentric case series with standardised follow‐up over prolonged periods of time, rendering valuable endpoints such as recurrence‐free survival (RFS) questionable. In addition, overall survival (OS) has been the primary endpoint reported in most studies and not disease‐specific survival (DSS). In addition, the absence of agreement on a strict cut‐off that may distinguish NET from NEC or TC from AC is also a major limitation for the use of Ki‐67 as a criterion for the histological subtyping of these tumours in clinical practice. Nevertheless, the WHO 2021 classification notes that Ki‐67 in lung NENs could help distinguish some LC from neuroendocrine carcinomas (NECs), with special reference to the pre‐operative setting.

Prognostic categorisation in NET or NEC is crucial for clinicians, since it defines two subgroups of patients with major differences in terms of prognosis and therapeutic management. The WHO 2021 classification mentions that highly proliferative carcinoids could be treated like large cell NECs (LCNECs). However, no role of the proliferative index for therapeutic response prediction has been validated, and overlap exists between NET and NEC categories that may challenge the therapeutic decision in some patients.

Several clinical studies have reported that a subgroup of LC may exhibit a high proliferative index, as defined by a mitotic count above 10/2 mm^2^ and/or a Ki‐67 above 20%, based on primary tumour or metastatic specimen analysis. This subgroup of patients represents around 5% of localised LC but up to 40% of metastatic LC. They behave both in terms of response to therapy and prognosis like LC and not NEC, and thus, these patients should be treated accordingly, contrary to the current WHO recommendations.^7^, ^8^ In that setting, clinical presentation, morphological criteria and the use of molecular markers may be considered. In such situations, wild type p53 and Rb expression favour the diagnosis of LC.^9^, ^10^

Recently, the randomised phase II MGMT‐NET trial reported a significantly higher overall response rate (ORR) with alkylating agents in foregut NETs with O6‐methylguanine‐DNA methyltransferase (MGMT) deficiency. However, most MGMT‐deficient NETs were of pancreatic origin, making the final role of MGMT determination in LC uncertain.^11^ Other subgroups of LC have been reported to be associated with a worse prognosis based on the presence of neoplastic nests or single cells in air spaces beyond the tumour edge (spread through airspaces, STAS), on the expression of a panel of proteins including the absence of orthopedia homeobox protein (OTP) and CD44 protein expression,^12^, ^13^, ^14^, ^15^ high telomerase reverse transcriptase mRNA expression,^16^ or single gene alterations or molecular subgroups based on genomic or multiomic profiling.^17^, ^18^, ^19^, ^20^, ^21^, ^22^ Lineage transition of LC to LCNE or SCLC has been reported, but its clinical relevance remains to be better understood.^23^ Finally, low expression of somatostatin receptor subtype 2 (SSTR2) has been correlated with poorer outcome in TC or AC LC.^24^ Based on this observation, but also on the prognostic role of somatostatin receptor imaging observed in other NETs, we consider PET SRI uptake not only a diagnostic tool but also a key imaging procedure that provides very relevant predictive and prognostic information in LC.

Nevertheless, the clinical relevance of all these biomarkers is weak in the absence of validation in distinct WHO (TC or AC) and TNM (early‐ versus late‐stage) annotated subgroups of LC and the use of robust endpoints.

#### 2.1.1 Recommendations

Despite the fact that Ki‐67 is not required for WHO histopathological classification, the panel recommends the routine determination of Ki‐67 to help discriminate carcinoids from NECs in cytological/biopsy specimens, and to identify a subgroup of carcinoids with more aggressive clinical behaviour (**4, A**). However, in the absence of clearly defined optimal cut‐off values, the final classification should consider clinical presentation, morphological criteria, functional imaging and the use of molecular markers. SSTR expression as detected by PET SRI should be considered as part of the prognostic classification of LC, as high uptake on all lesions is associated with a more favourable outcome.

Abnormal expression of Rb and p53 is typical of lung NECs, but normal expression does not completely rule out this diagnosis. Their use is not mandatory for the classification of LC but may help discriminate AC from LCNEC in some difficult cases (**4, B**). No other tissue markers are routinely recommended in clinical practice.

#### 2.1.2 Specific considerations regarding thymic carcinoids or MEN1 status?

Somatic *MEN1* mutation is a potential adverse prognostic indicator in sporadic LC, but an LC primary as part of the MEN1 syndrome has not been assigned any negative prognostic role by contrast with ThC.^17^, ^22^, ^25^

ThC are classified according to 2021 WHO classification of *Thoracic Tumours*; most of them are atypical and the prognostic role of the WHO classification or Ki‐67 is controversial. A high number of highly proliferative AC have been recently reported in advanced ThC, further underlying the more aggressive biology of this NET primary.^26^

### 2.2 Imaging

Q2: What is the most appropriate imaging for accurate TNM staging, and is there a need for a dedicated TNM classification?

The *Union for International Cancer Control (UICC) TNM Classification of Malignant Tumours* recommends staging LC according to the same criteria used for carcinomas. The accuracy of this classification to predict outcomes of TC and AC, however, remains to be properly assessed. Recently, the 9th TNM edition of the IASLC has been published which mainly proposes a revised pN classification for lung cancer: thus, with regard to N2 status, this is divided into N2a (single site) and N2b (multiple sites).^27^ In metastatic disease, tumour burden and tumour growth rate (TGR) are additional parameters that should be taken into consideration to stratify patient prognosis due to prolonged survival of a subgroup of patients.

Although a significant difference in outcomes for grouped stages has been observed, with a 10‐years DSS ranging from 96% for stage I to 59% for stage IV for TC, no significant differences were observed between substages (i.e., IA vs. IB), and similar results were found for AC.^28^ In addition, a single metastasis may not exist in LC, and multiple nodules in the lung may reflect multiple primaries, with indolent behaviour rather than the poorer prognosis inherent with distant metastases, consistent with the good prognosis reported for LC patients categorised as having isolated lung metastases.^29^, ^30^, ^31^ In cases of multiple LC nodules, the diagnosis of diffuse idiopathic pulmonary neuroendocrine cell hyperplasia (DIPNECH), MEN1 syndrome or sporadic lung multiple primaries should be considered as an alternative to lung metastases, especially when no disease progression is observed.^30^, ^32^, ^33^ Interestingly, all these conditions have been associated with relatively indolent behaviour, suggesting they may be classified as stage I rather than IV as currently established in the lung UICC classification.^34^ Therefore, the relevance of the M1b category, as defined by single metastasis, or the T3 or T4 categories, when based on ipsilateral or contralateral lung nodules, is questionable.

After curative surgery, N status, subclassified in pN0‐N1‐N2 categories, is considered the most relevant prognostic parameter, but standardised N staging is rarely performed in real life.^35^ In such a context, appropriate classification of the T category remains important: retrospective studies suggest the prognostic relevance of the 2, 4 or 7 cm primary size cut‐offs.^28^, ^30^, ^35^, ^36^

The most common sites of LC haematogenous metastases are the liver (51–75% of cases) and bone (16–45% of cases). Brain metastases are less frequent (1.5–5%), although more common in LC, particularly AC, than in NETs from other primary sites. Lung metastases are rare (10% of cases), and because they are difficult to distinguish from multifocal LC (e.g., DIPNECH), their true frequency remains unclear. Finally, the diversity of metastatic sites is notable, including skin, pancreas, peritoneum, breasts, orbits, ovaries and others.^29^, ^37^

#### 2.2.1 Recommendations

At present, UICC TNM staging of LC is advised, although only the pN category may be considered validated in this setting, whereas the pT and pM categories need further specific assessment. The panel recommends expert centres to collect the exact pT size and detailed locoregional extent to surrounding structures, the pN status with the exact number and locations of positive and harvested lymph nodes, and the precise location and extent of metastatic sites.

Proper TNM staging of LC should be based on a combination of multiphase contrast‐enhanced CT (with late arterial and portal phases) and somatostatin receptor imaging positron emission tomography (SRI‐PET) (**2, B**). Liver magnetic resonance imaging (MRI) of the abdomen including dynamic acquisition and diffusion‐weighted sequences of the liver, preferably with hepatospecific contrast, can be useful for liver metastasis detection in the case of suspicion on initial assessment or before liver‐directed therapy (**2, B**). A brain MRI is not routinely recommended in the absence of clinical suspicion (**3, B**). ^18^F‐FDG‐PET‐CT may be considered in selected patients, mainly in cases of low or absent uptake on SRI‐PET (**3, B**), but no prognostic role of ^18^F‐FDG‐PET‐CT has been validated in LC.^38^ Recent studies have suggested that SRI‐PET underestimates disease extent in up to 50% of patients.^39^, ^40^

#### 2.2.2 Specific considerations regarding thymic carcinoids or MEN1 status

About 25% of ThC occur in the context of MEN1 syndrome and, consequently, screening of ThC is recommended as part of the initial workup of these patients. In addition to ThC, MEN1 is characterised by the occurrence of parathyroid, pancreatic islet, and anterior pituitary tumours, which should be screened for in this context and not misdiagnosed as metastatic sites of disease. Some patients may also develop adrenocortical tumours, meningiomas, facial angiofibromas, collagenomas, and lipomas, and there is probably an increased incidence of breast cancer.

ThC have a higher rate of R1 resection and metastatic disease, with common pleural and pericardial involvement.^26^ If surgery is indicated, MRI of the mediastinum is recommended in addition to CT to assess tumour reseability, which should be performed in expert centres (**4, C**). Dual SRI‐PET and ^18^F‐FDG‐PET‐CT avidity evaluation is also recommended^26^ (**4, C**).

### 2.3 Biology

Q3: Which biomarkers and when should be used in daily practice?

Historically, serum chromogranin A (CgA) was routinely measured in all patients, while other specific biomarkers, such as 5‐hydroxyindoleacetic acid (5‐HIAA), adrenocorticotropic hormone (ACTH), urinary‐free cortisol (UFC), growth hormone‐releasing hormone (GHRH), and insulin‐like growth factor 1, can be assessed based on the presence of functioning syndromes. In patients with advanced LC, baseline measurement of CgA is recommended due to its potential prognostic value, serving as a marker of secretory tumour burden in metastatic disease.^29^, ^41^ However, given its limited sensitivity and specificity, CgA measurement is not recommended as a screening tool for diagnosis or for monitoring during follow‐up, including patients that undergo a complete surgical resection.

In addition to patients with carcinoid syndrome (CS), routine assessment of urinary 5HIAA is now recommended for all metastatic LC patients, as elevated levels have been reported in asymptomatic metastatic LC patients, and are associated with an increased risk of carcinoid heart disease (CHD).^42^, ^43^ In patients with elevated 5HIAA levels, measurement of NT‐proBNP may be useful for screening and monitoring CHD.

Measurement of UFC is primarily indicated in patients with Cushing syndrome (CuS), which may occur in patients with localised or advanced LC, and could determine prognosis. Similarly, growth hormone‐releasing hormone (GHRH) and insulin‐like growth factor 1 should be assessed based on the presence of acromegaly, although this may alternatively indicate the presence of multiple endocrine tumour syndrome.

Progastrin‐releasing peptide is an emerging new biomarker of potential use in the diagnosis and monitoring of lung NENs, but further validation is needed before routinely recommending its use in clinical practice.^44^

A multianalyte molecular assay, the *NETest* (51 transcripts; NET test), is more sensitive than CgA in distinguishing patients with NET from healthy individuals, but has low specificity precluding its use as a diagnostic biomarker.^45^ There are also data regarding its ability to identify patients with progressive disease and to predict response to various therapies, but validation in larger prospective and adequately controlled patient cohorts is required before its general use in clinical practice.^46^ Circulating tumour DNA (ctDNA) (liquid biopsy) offers a new tool to molecularly characterise advanced disease that may play an increasing role in identifying potentially druggable genetic alterations, hereditary syndromes or other future biomarkers.

#### 2.3.1 Recommendations

The panel currently recommends the measurement of CgA and 5‐HIAA in all metastatic LC patients, whatever their functioning status at baseline. In metastatic LC patients, during follow‐up, even in asymptomatic patients we recommend the monitoring of 5‐HIAA levels to ensure they remain below 3–5 the UNR at least once a year (**4, B**). Plasma 5‐HIAA may replace urinary 5‐HIAA as it has similar diagnostic performance with improved patient convenience.^47^ UFC should be measured in cases of suspected CuS and monitored during follow‐up if increased (**4, A**).

#### 2.3.2 Specific considerations regarding thymic carcinoids or MEN1 status

Dedicated hormonal work‐up according to MEN1 guidelines should be performed in these patients.

In ThC, CuS is more frequent than in LC and requires a dedicated clinical and biological screening at diagnosis, and also in case of newly diagnosed hypokalaemia or diabetes in these patients.^26^

### 2.4 Therapy—Surgical aspects

Q4: When can observation or wedge/segmentectomy be considered a safe approach in localised lung carcinoid patients as compared to standard lobectomy plus lymph node dissection?

Anatomical surgical resection (segmentectomy, lobectomy, bilobectomy) with standardised routine lymph node dissection is recommended in most patients. The *European Society of Thoracic Surgeons* lymph node resection guideline (a minimum of six nodal stations—three hilar and three mediastinal, always including the subcarinal station) must be followed for adequate tumour pathological staging.^48^, ^49^ Preservation of as much lung parenchyma as possible is critical. Thus, pneumonectomy is contraindicated except in very rare situations that must be always discussed in expert centres specialised in broncho‐plastic procedures (e.g., sleeve resections or bronchial resections without lung sacrifice), which constitute the preferred option for suitable centrally‐located tumours. The choice of open surgery or minimally invasive approach (VATS—video assisted thoracic surgery—or robotic assisted thoracic surgery) will depend on tumour size and location, pre‐operative cytology/histology and the surgeon's experience.

Surgical resection has been associated with improved OS in LC compared with observation,^50^, ^51^ and with improved DSS^52^, ^53^, ^54^ as well as superior OS relative to expected population‐based estimates.^55^ However, in TC, observational cohorts have shown excellent outcomes, with a 5‐ and 10 year‐DSS of 88%–89% and 85% for stage I TC and a 10‐year DSS of 84% for T1a TC,^1^, ^53^, ^54^ suggesting that non‐operative management may be appropriate for carefully‐selected patients. In contrast, the poor prognosis of AC makes surgery the unequivocal standard of care for these patients. Due to the lack of prospective trials, limited granularity in the definition of sublobar resections (wedge vs. segmentectomy), and heterogeneous endpoints, the optimal extent of surgery remains debated. The benefit of lobectomy with systematic lymph node dissection has been best demonstrated in AC.^36^, ^56^ By contrast, room for discussion exists in stage 1 or T1a TC since the benefit of anatomical resection (lobectomy or segmentectomy) over wedge is reported in some^57^ but not all series.^50^, ^52^, ^58^, ^59^

Due to the demonstrated prognostic value of nodal status, the correlation between tumour size and lymph node involvement, and the poor accuracy of preoperative nodal staging,^35^ primary tumour size may help identify which patient may most likely benefit from oncological resection. Consistent with this, the marked decline in OS reported for TC >2 cm and positive LN, and of 10‐year DSS reported for TC >4 cm suggest room for discussion in other stage 1 TC and support a more individualised surgical approach in early‐stage TC.^28^, ^36^

In addition, hormonal syndromes should be well controlled in functioning tumours prior to and during the operation, best performed in NET expert centres with experienced anaesthesiologists.

#### 2.4.1 Recommendations

The panel recommends anatomical surgical resection with standardised routine lymph node dissection in most patients (**3, A**), but alternative approaches may be considered in specific clinical scenarios and in patients with limited lung functional reserve or comorbidities (**4, B**). Pneumonectomy must be avoided as much as possible (**4, B**).

Alternative strategies may be considered in specialised centres for the certain conditions associated with a more indolent clinical course, typically stage 1 TC patients, or in patients with competing survival risks: (a) LC patients presenting within DIPNECH, who are characterised by multiple primaries and in whom altered lung function may constitute the main issue; (b) MEN1 patients, who are characterised by multiple NETs that may include other synchronous or metachronous tumours that are potentially more aggressive than the usual lung primaries (duodeno‐pancreatic or thymic NETs); (c) multifocal presumably sporadic LC primaries, which remain a poorly‐defined entity; and (d) patients with comorbid conditions that challenge the 5 or 10‐year specific LC survival. Alternative options may include watch‐and‐wait follow‐up strategies in asymptomatic patients in the absence of morphological progression or somatostatin analogues (SSA) therapy upon disease progression or upfront in symptomatic patients, or ablative therapy such as radiofrequency ablation or stereotactic body radiation therapy (SBRT) (5, C). Lung‐sparing segmentectomy or non‐anatomical wedge resection may also be considered to confirm and definitively classify LC according to WHO criteria (5, C).

#### 2.4.2 Specific considerations regarding thymic carcinoids or MEN1 status

MEN1 patients may present multiple NET primaries with different malignant potential that require expert clinical judgement for adequate management.

ThC are more aggressive than LC and surgery represents the treatment of choice. Sternotomy, sometimes along with anterior thoracotomy (combined approach), or thoracotomy are the commonest surgical approaches to achieve a complete (R0) tumour resection. As recommended by most recent *International Association for the Study of Lung Cancer* (IASLC) guidelines, superficial and deep lymph‐node resection must always be performed to achieve a correct pathological tumour stage, which may guide subsequent adjuvant therapy.^60^, ^61^

Q5: Which is the best therapeutic strategy in case of R1 resection for a localised lung carcinoid?

The impact of resection status has been poorly investigated in LC and its prognostic role is largely unknown.^35^, ^36^ Complete resection is reported in more than 90% of LC, but this rate may be lower in patients undergoing sublobectomy.^35^, ^36^, ^58^ The classification of R status for lung cancer has been recently revised by the IASLC though its applicability for LC remains to be established.^62^ Its management mainly depends on expected prognostic outcome, as well as feasibility and risks associated with reintervention in each patient.

The risk of R1 resection mainly depends on the following factors: (a) tumour location (higher for centrally‐located tumours); (b) the presence of large/bulky lymph‐nodal involvement; and (c) primary tumour local aggressiveness and invasion of adjacent anatomical structures. The most recent recommendation of the IASLC defines R0 status by ‘no identifiable tumour remaining, negative surgical margins, adequate node assessment and, highest node station is negative’.^62^ Intraoperative frozen section analysis of margins is recommended, especially in central tumours where free margins are more difficult to achieve. Tissues at risk of direct invasion by the tumour include the pericardium, diaphragm, parietal pleura and chest wall, superior vena cava, trachea, oesophagus and other great vessels.

#### 2.4.3 Recommendations

The recommended therapeutic strategy for R1 resected tumours (as defined by microscopically positive margins) depends on: (a) post‐operative WHO subtype (TC vs. AC) and pTNM stage; (b) site(s) of incomplete resection; (c) feasibility of a reintervention and risk of postoperative complications, especially on respiratory function; and (d) presence of a functioning syndrome such as CuS.

A reintervention should be considered if feasible and the patient tolerates major surgery (usually lobectomy/bilobectomy), especially for AC, bulky N positive TC or CuS (**5, C**). As for other primary lung cancers, SBRT is a therapeutic alternative in patients not fit for reintervention (**5, C**). Radiotherapy is not recommended in case of large mediastinal lymph nodes that have not been completely resected due to the high risk of severe complications on the cardiac muscle, oesophagus and/or pulmonary parenchyma. R1 status without additional therapy may be considered acceptable for stage 1 TC in the same settings as mentioned in Q4.

#### 2.4.4 Specific considerations regarding thymic carcinoids or MEN1 status

In patients with MEN1, lung parenchyma‐sparing strategies are recommended and reintervention should be discussed case‐by‐case based on WHO and pTN status, and on extent and tumour control of other NET primaries (**5, C**).

Incomplete surgical resection, which constitutes a strong negative prognostic feature, is more common in ThC (R1‐2 in up to 28%),^63^, ^64^ and should be avoided by all preoperative means. This includes appropriate preoperative staging, surgery by an expert surgeon, and routine reoperation should be discussed in all patients.

Q6: Role of locoregional treatment on primary tumour or metastatic sites?

Locoregional therapies (LRT) have not been well explored in LC but may be used for symptomatic or tumour control, including hormonal syndrome control and the prevention of local disease‐related complications, and could delay the need for the initiation of systemic therapies.

One study reported up to 65% of LC patients received at least one form of LRT during the course of metastatic disease.^29^ While these interventions are primarily palliative, a locally curative intent may be considered in certain cases to prevent disease‐related local complications. The efficacy of SBRT for primary LC tumours has not been established in prospective studies. However, SBRT may be preferred over conventionally fractionated radiotherapy.^65^

In bronchial tumours, bronchoscopic debulking may be considered to restore airway patency, thereby facilitating surgical resection^66^ or allowing surgery to be deferred^67^ Endobronchial treatment or even primary surgery may also be used for symptom relief in cases of atelectasis, air trapping, debilitating cough, obstructive pneumonia, or haemoptysis in selected patients with metastatic LC.

Whether oligometastatic LC exists remains an open question. Indeed, microscopic metastatic dissemination has been shown to be underestimated in advanced NETs.^68^, ^69^

The higher frequency of extrahepatic metastatic sites of disease makes it more difficult to position liver‐directed LRT than in digestive NETs, but it is still of value, particularly to control hormonal secretion in functioning tumours.^70^, ^71^ In a recent series of 202 patients including 44 LC, trans‐arterial chemoembolisation provided 45% ORR according to RECIST and 72% with a significant clinical response.^72^

The prevalence of bone metastasis in NETs varies from 7% to 42%, and appears to be higher in LC than in NETs from other origins.^29^, ^73^ Most common skeletal‐related events (SRE) are bone pain (62%), spinal cord compression (10%) and pathological fracture (9%).^73^, ^74^ Radiotherapy, thermo‐ablation, cryoablation or surgery are frequently used to prevent or treat SRE. Osteoclast inhibitors are approved for the prevention of SREs in adults with advanced malignancies involving bone metastasis, and can also be used in this setting.

#### 2.4.5 Recommendations

LRT should always be considered to treat or palliate hormone‐ or tumour‐bulk‐related symptoms or local complications, or in patients for whom a delay of systemic therapy is a clinically relevant goal, despite the lack of prospective studies in LC. LRT approaches, both on the primary tumour and metastatic sites, should be evaluated case by multidisciplinary teams which should include thoracic, abdomen and bone‐dedicated surgeons, interventional radiologists, and radiation oncologists (**5, B**). Specialised anaesthesiologists are required as part of the NET multidisciplinary team. These procedures should be integrated as part of a sequential multimodal strategy in the continuum of care of these patients.

#### 2.4.6 Specific considerations regarding thymic carcinoids or MEN1 status

These recommendations are also applicable for MEN1 and ThC patients, although for the latter, the more frequent pleural dissemination may reduce the relevance of LRT.

Q7: Is there a role for adjuvant therapy in M0R0 resected typical and atypical carcinoids?

The available literature suggests no benefit from adjuvant therapy in TC and AC,^75^, ^76^ although no randomised trials have been conducted to date to properly address this question. However, it may be considered after multidisciplinary discussion in selected fit patients with particularly high risk of relapse, such as AC with high proliferative index and/or N2 disease.^36^ Because distant metastases predominate at recurrence, systemic intervention should be discussed, although there is no consensus on the best regimen (alkylating‐ or platinum‐based chemotherapy). SSTR expression is lower in the LC subgroup with higher risk of relapse (mainly, AC), and in any case, adjuvant peptide receptor radionuclide therapy (PRRT) is currently not advised in the absence of visible residual disease on post‐operative SRI. SSA or everolimus is also not recommended in the adjuvant setting, since they are mainly cytostatic treatments.

Two large randomised controlled phase 3 trials of adjuvant postoperative radiotherapy in resected N2 positive NSCLC showed no difference in RFS and OS at 3 years.^77^, ^78^ No specific data are available in LC, but benefit is not expected as it is a less radiosensitive disease.

#### 2.4.7 Recommendations

Adjuvant therapy is not indicated in the majority of patients with completely resected (R0) localised disease (**4, C**). pN2 AC patients, particularly those with higher proliferative rates, constitute the target population for systemic adjuvant therapy. Both platinum‐ or alkylating‐based regimens may be considered (**4, C**). MGMT status may help refine the decision in selected patients. Oxaliplatin is preferred when platinum‐based chemotherapy is contemplated due to its more favourable safety profile.

#### 2.4.8 Specific considerations regarding thymic carcinoids or MEN1 status

After resection in patients with MEN1 no adjuvant treatment is advised, since radical treatment is not the purpose of surgery in LC patients (**5, C**). Case‐by‐case discussion is recommended for additional local and/or systemic options in stage III ThC following R0 resection (**5, C**).

### 2.5 Therapy—Syndrome control

Q8: How should Cushing syndrome be managed in the preoperative or palliative settings in lung and thymic carcinoid patients?

The importance of managing CuS is linked to complications caused such as sepsis, cardiovascular disease, metabolic disorders, and thromboembolic events, which should be actively screened and treated.^79^, ^80^, ^81^, ^82^

There are a variety of options to treat CuS but there is no consensus on the best strategy due to the lack of specific large‐scale studies on LC adjusted for tumour characteristics. Metyrapone and ketoconazole are recommended as first‐line therapy. Osilodrostat constitutes an alternative option.^83^, ^84^ In patients with acute emergency situations administration of etomidate in intensive care unit could be an option or, in patients with refractory CuS and metastatic LC requiring active anti‐tumour therapy, early bilateral adrenalectomy could be considered. Therapeutic approaches in patients with CuS should balance risks associated with hypercortisolism and tumour characteristics (TNM and WHO classifications). Anti‐tumour management must pay attention to the specific complications of CuS, including the increased risk of sepsis with chemotherapy or everolimus, or of thromboembolism with antiangiogenic drugs. Attention should also be paid to potential drug–drug interactions (DDI), which includes targeted agents (i.e., everolimus>multikinase inhibitors). Ketoconazole poses the highest risk of DDI as it is a strong inhibitor of CYP3A4. Osilodrostat also needs, although to a lesser extent, CYP3A4 interaction monitoring (mild CYP3A4 inhibitor). Monitoring of QTc prolongation related to ketoconazole or osilodrostat is particularly relevant in case of hypokalaemia or the concomitant use of multikinase inhibitors. Attention should also be paid to the fact that some of these drugs lead to an accumulation of precursors which may interact with some cortisol assays.

#### 2.5.1 Recommendations

**FIGURE 1:**
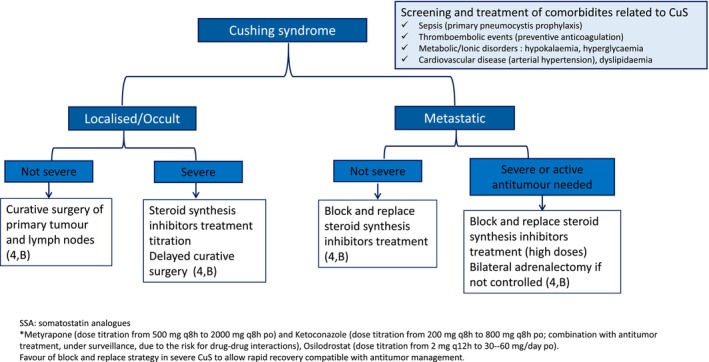
Management algorithm for Cushing's syndrome (CuS) of lung or thymic carcinoids.

Diagnosis and characterisation of CuS requires dedicated endocrinological expertise as part of the NET MDT board. In case of severe CuS and localised LC, surgical resection may be delayed a few months and CuS control with steroid inhibitor titration should be prioritised (**4, B**) (Figure 1). Upfront surgery may only be considered in localised LC with non‐severe CuS. In patients with advanced and progressive LC, antitumor intervention should not be significantly delayed and the steroid inhibitor block and replace therapies of CuS are generally recommended (**4, B**). Steroid inhibitors with rapid onset of action and lower risk for DDIs are preferred (metyrapone or osilodostrat) (**4, B**). Bilateral adrenalectomy may be considered in severe CuS resistant to steroid inhibitors in metastatic LC (**4, B**). In all cases, prevention of comorbidities and education on adrenal insufficiency management is required.

#### 2.5.2 Specific considerations regarding thymic carcinoids or MEN1 status

In MEN1 patients, CuS is rare and different causes of CuS should be carefully investigated in a specialised unit (**5, B**).

CuS is more frequent in ThC than in LC.^85^ It commonly reveals advanced disease. The same strategy described for LC is proposed for ThC.

Q9: How should carcinoid syndrome be managed in the non‐curative setting?

CS is present in 1%–5% of patients with LC at diagnosis, but this rate increases to 15%–40% in patients with liver metastases.^29^, ^86^, ^87^ CS symptoms most commonly include diarrhoea and flushing, but patients may have more atypical presentations including lacrimation, rhinitis, asthma and palpitations. In properly screened populations, up to 20% of patients will also develop CHD.^29^ Occasionally CS may derive solely from a localised LC and may therefore cause left sided heart valvular disease. Screening for CHD by a dedicated cardiologist with echocardiography is mandatory in all patients with CS and/or increased 5‐HIAA levels even if asymptomatic.^88^, ^89^

CS is treated as in gastro‐entero‐pancreatic (GEP) NETs, as dedicated trials focusing on functioning LC are lacking. The goals of CS treatment are to limit symptoms and their impact on patients' daily activities and to lower urinary 5‐HIAA levels and prevent CHD or slow‐down its worsening. Recommended first‐line treatment is SSA, octreotide LAR 30 mg or lanreotide autogel 120 mg, both administered every 28 days. SSA therapy can achieve symptomatic relief in 65%–75% of patients, biochemical responses in 40%–50%, and have demonstrated antiproliferative activity in randomised trials performed in low proliferative GEP‐NET patients.^88^, ^90^, ^91^, ^92^ Second‐line options should be discussed if no improvement or deterioration of CS. Prospective randomised trials in refractory CS patients have shown that monthly administration of pasireotide 40 mg or octreotide 60 mg were able to provide an appropriate symptomatic control in about 20%–27% of patients.^93^ In addition, telotristat ethyl, an oral tryptophan hydroxylase inhibitor, was found to improve diarrhoea in 42%–44% of patients with refractory CS despite SSA, and to induce a 30% u5‐HIAA level reduction in 78%–87% of patients.^94^, ^95^ However, the primary tumour origin of patients enrolled in this trial was not reported, and specific CS analysis in LC patients is thus lacking which limits final conclusions. Other randomised prospective studies have reported the benefit to add interferon, everolimus or PRRT with ^177^Lu‐Dotatate to SSA on CS symptom control.^96^, ^97^, ^98^ Moreover, multiple organ‐directed LRT including cytoreductive surgery may be a very effective treatment strategy to improve CS control in patients with liver‐dominant disease (see Q6). Finally, any therapeutic strategy that can induce tumour shrinkage or improve growth control may be of some value in terms of CS control (i.e., FOLFOX), although evidence is limited. Of note, 5‐HIAA response is scarcely reported in metastatic NET trials and all these options remain palliative on CS control.

#### 2.5.3 Recommendations (Figure 2)

**FIGURE 2:**
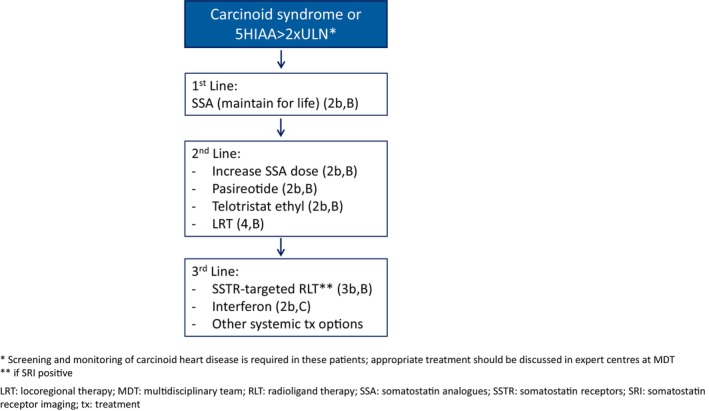
Management algorithm for carcinoid syndrome (CS) of lung or thymic carcinoids.

SSA is the treatment of choice for first line therapy of CS (**2b, B**). Short‐acting SSA can be given as breakthrough medication (**3b, B**). In refractory cases, as defined by persistent diarrhoea (>3 bowel movements per day), debilitating flushes and/or 5‐HIAA levels>3–5xUNR, different options should be considered early in the course of the disease (within 3–6 months), based on tumour burden, growth rate, SSTR expression and sites of metastasis. Increasing doses of SSA (**2b, B**), telotristat ethyl (**2b, B**), or pasireotide (**2b, B**) are generally the preferred second‐line treatment options in the context of refractory CS and stable disease. PRRT (**3b, B**), LRT (**4, B**) or interferon (**2b, C**) may be more suitable options when high tumour burden requires more active antitumor control. Other systemic therapies (everolimus or chemotherapy) may be required for tumour control in progressive tumours. Exacerbation of CS induced by LRT or PRRT has been described and should be anticipated and adequately prevented and managed^99^, ^100^, ^101^, ^102^ (**4, B**). Although optimal treatment sequencing is still a matter of debate, active surveillance and dedicated anti‐secretory management is recommended even in patients with non‐progressive LC.

#### 2.5.4 Specific considerations regarding thymic carcinoids or MEN1 status

CS is very rarely associated with MEN1 or ThC^26^, ^103^ and should be managed as in LC.

### 2.6 Therapy—Tumour control

Q10: What is the recommended first‐ and second‐line therapy to control slowly progressive advanced typical and atypical carcinoids?

Discussion on the best indication of systemic interventions in LC is mainly based on the results of extra‐pancreatic NETs from phase II–III trials, including LC/ThC and GI NETs, and a few dedicated thoracic NET phase II–III trials. All current medical options are palliative. To date, advanced LC prognosis still drives guidance. Median OS of metastatic LC has been reported to be up to 81 months but may widely range from 33 to 105 months according to prognostic factors.^29^, ^104^, ^105^ This heterogeneous outcome is best refined by the analysis of TGR during the first months of follow‐up. Patients with TC, slow TGR and low tumour burden constitute the best prognostic subgroup in which safety of interventions and preservation of quality of life during initial management is the main therapeutic objective. The panel defined the safety of drug interventions as absence of drug‐related mortality and long‐term adverse events (AEs).

SSA have been shown to be the safest drug intervention in advanced LC providing significantly improved PFS as compared to placebo in low grade and slowly progressive advanced GEP NETs.^90^, ^91^ The SPINET trial, a phase III trial comparing lanreotide versus placebo in SSA‐naïve advanced LC, stopped prematurely (77 patients enrolled) due to slow accrual likely due to off‐label SSA use as standard of care in these patients. The mPFS was 16.6 months in the SSA arm versus 13.6 in the placebo arm (HR 0.94, *p* = .866), although the study was underpowered to demonstrate statistical significance of the observed difference. The benefit seemed greater for TC than AC.^106^, ^107^ In the LUNA trial, that enrolled 124 patients (32% TC and 68% AC) with advanced LC (95%) or ThyC (5%), that were randomised to receive pasireotide, everolimus or both drugs, pasireotide monotherapy showed preliminary evidence of activity, with a 9‐month disease control rate of 39% and median PFS of 8.5 months, but a debatable synergistic effect when combined with everolimus.^87^, ^108^

Second‐line treatment options for slowly progressing tumours may consider high‐dose SSA therapy as in the CLARINET Forte trial or the control arm of the NETTER‐1 or NETTER‐2 trials, although specific data in LC is lacking. LRT, most commonly targeting liver or bone disease, may also be a valuable treatment option in this setting to palliate local or hormonal symptoms, prevent local complications (i.e., SRE), or to control oligo‐progressive disease. If these options fail, pasireotide may be an option as discussed above. In patients with more rapidly or overt disease progression, systemic treatment options may be considered, such as everolimus, cabozantinib, alkylating agents in dMGMT patients or PRRT in SRI positive tumours, as will be discussed in the next section. New options or alternatives, such as lower doses of established options in NET, could be explored in the setting of indolent disease, with the aim to avoid long term AEs. Further research is needed to confirm that this strategy does not compromise efficacy.

#### 2.6.1 Recommendations (Figure 3)

**FIGURE 3:**
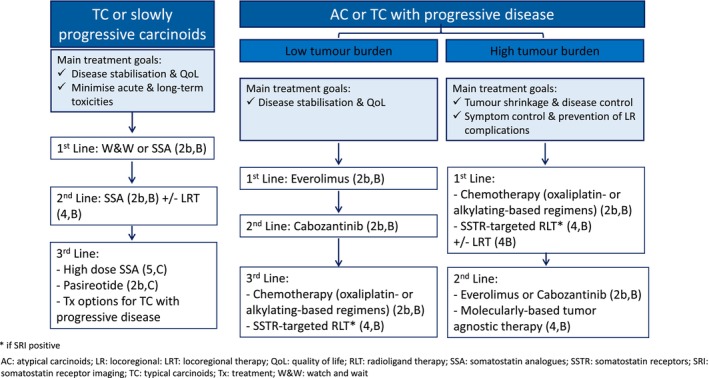
Systemic therapy for advanced lung (LC) or thymic carcinoids (ThC).

SSAs are the recommended first‐line treatment in patients with SST‐positive TC and/or slowly progressing advanced LC with low tumour burden (**2b, B**). A watch‐and‐wait strategy can be an option for low tumour burden, slowly growing and asymptomatic tumours (**5, B**). On progression on standard doses of SSA, high doses of SSA (**5, C**) or pasireotide (**2b, C**) may be considered in low tumour burden, slowly progressive LC. LRT (**4, B**) or other systemic treatments (**2b, B**), as discussed in Q11, may be considered in high tumour burden patients or more rapidly growing tumours. Lower exposure, less toxic therapeutic strategies should be investigated in this setting.

#### 2.6.2 Specific considerations regarding thymic carcinoids or MEN1 status

The strategy in MEN1 or ThC is similar (**5, C**). Expression of SSTR in ThC can be lower than in other NETs, which has an impact for PRRT eligibility.^109^

Q11: What is the recommended first‐ and second‐line therapy to control advanced atypical carcinoids or progressing typical carcinoids?

Systemic treatment options for LC are limited and include everolimus, angiogenesis inhibitors, SSTR‐targeted PRRT and chemotherapy. Optimal treatment sequence is a matter of debate, as head‐to‐head comparisons and predictors of response are not available in this setting and recommendations are made based on patient and tumour features, treatment goals and best available evidence in GEP NET patients mainly.

Everolimus was approved by the Food and Drug Administration (FDA) and European Medicines Agency for non‐functioning digestive and LC based on RADIANT‐4 results, a randomised controlled trial (RCT) that included 302 pretreated patients (including 90 LC) with rapidly progressive tumours. Subgroup analysis of LC patients enrolled in this trial demonstrated a significant improvement of PFS for everolimus‐treated patients as compared to those treated with placebo (9.2 vs. 3.6 months, HR 0.50, 95% CI, 0.28–0.88). Most common grade 3–4 treatment‐related AEs were stomatitis (9%), diarrhoea (7%), infections (7%), anaemia (4%), fatigue (3%) and pneumonitis (1%). Rash and peripheral oedema are also common but generally mild and manageable. A dose decrease due to AEs was required in 67% of patients, and drug withdrawal in 12%.^110^, ^111^ The RADIANT 2 trial also investigated everolimus versus placebo, both combined with SSA, in advanced functioning, mostly extra‐pancreatic (ep) NETs (including 44 LC). This trial also showed a clinically meaningful increase in PFS of 5.1 months for everolimus‐treated patients in the entire trial population (16.4 vs. 11.3 months, HR 0.77, 95% CI 0.59–1.00), but it did not reach statistical significance (*p* = .026 vs. the prespecified threshold of *p* = .0246), making the positioning of everolimus in slowly growing functioning NETs somewhat weaker. Subgroup exploratory analysis showed a similar beneficial trend in patients with LC (13.6 vs. 5.6 months, HR 0.72, 95% CI, 0.31–1.68).^98^, ^112^

Multikinase inhibitors (MKI) have demonstrated activity in LC in three phase III trials (SANET‐ep, AXINET and CABINET trials) leading to the approval of surufatinib in China and, more recently (2025), cabozantinib in the USA (FDA) and Europe (EMA) for the treatment of patients with epNETs following progression to at least a prior line of systemic therapy other than SSA.^113^, ^114^, ^115^ Cabozantinib is a TKI targeting VEGF‐R, c‐MET, AXL and RET. The CABINET trial was a RCT that enrolled 394 G1‐3 pancreatic and ep‐NETs progressive to at least one prior line of FDA‐approved therapy (everolimus for LC), that were randomly allocated 2:1 to receive cabozantinib or placebo.^115^ The study was terminated early based on compelling efficacy data upon second interim analysis for the epNET cohort (*n* = 203, of which 39 LC (19.2%)) based on PFS. The mPFS was significantly improved with cabozantinib in these patients (8.4 vs. 3.9 months, HR 0.38, 95% CI, 0.25–0.59). ORR with cabozantinib was 5% in ep‐NETs Subgroup analysis of LC patients enrolled in this trial demonstrated a significant improvement of PFS for cabozantinib‐treated patients as compared to placebo (8.2 vs. 2.7 months, HR 0.19, 95% CI, 0.06–0.54). ORR with cabozantinib was 6% in LC.^116^ Most common treatment‐related AEs in this cohort of grade 3 or higher were hypertension (21%), fatigue (13%) and diarrhoea (11%). Grade 5 events deemed to be at least possibly drug‐related occurred in 4 (2.9%) patients. Dose reductions were required in 66% of patients and drug withdrawal for AEs in 31%. The other two phase 3 trials (AXINET, SANET‐ep) showed efficacy of MKIs in less heavily pretreated LC patients, more specifically, in patients not pretreated with everolimus, although only the SANET‐ep trial met the trial primary endpoint.

Chemotherapy can also be an option for LC mainly based on retrospective studies. With temozolomide‐based chemotherapy, ORR of 18%–30% and PFS of 9–13 months have been reported.^29^, ^117^ Temozolomide plus lanreotide showed a low ORR (2.5%) and a mPFS of 37.1 months in a prospective phase II study conducted in LC/ThC patients with progressive disease within 1 year prior to study entry.^37^ Oxaliplatin‐based chemotherapy is the second most studied chemotherapy regimen.^118^, ^119^ A study that included 45 patients with metastatic LC treated with either GEMOX or FOLFOX reported an ORR of 20%, a mPFS of 15 months, and a mOS of 34 months.^118^ Tumour MGMT status may help in the choice of chemotherapy regimen as shown in the MGMT‐NET trial.^11^ This was a biomarker‐stratified randomised phase 2 trial that enrolled 105 patients with advanced grade 1–3 NETs (35 LC) that were assessed at baseline whether they were MGMT proficient (*p*) or deficient (*d*). Patients were randomly assigned 1:1 for p‐MGMT or 2:1 for d‐MGMT NETs to receive either alkylating (*n* = 62) or oxaliplatin (*n* = 43) based chemotherapy. Alkylating agents proved more effective in d‐MGMT NET patients, with an ORR of 52.9% versus 11.5% and a mPFS of 14.6 versus 11.3 months in *d*‐ versus *p*‐MGMT tumours, respectively. MGMT status did not seem to affect efficacy of oxaliplatin‐based chemotherapy. Thus, when MGMT status is unknown, oxaliplatin‐based chemotherapy may be preferred.

Finally, PRRT with ^177^Lu‐DOTATATE can be an interesting therapeutic approach based on extrapolation from the NETTER‐1 and ‐2 trials in GEP‐NETs. However, expression of SSTR in LC is lower, and solid evidence from randomised trials in LC is lacking. PRRT results in LC are based on retrospective series and one prospective study, which reported ORR ranging from 15% to 33% in the largest series.^120^, ^121^ A phase II study (*n* = 34) showed tumour control in 80% of patients with TC and 47% of AC, and a mPFS of 20.1 and 15.7 months, respectively.^120^ The first randomised phase III trial (COMPETE trial) comparing PRRT with a targeted therapy, namely everolimus, demonstrated a significant improvement in PFS of PRRT over everolimus in G1‐2 GEP‐NETs.^122^ Similarly, the first randomised phase II trial (OCLURANDOM trial) has demonstrated significant improvement in 12‐month PFR comparing PRRT versus sunitinib in pancreatic NET.^123^ No survival benefit is currently demonstrated with any available option. RCTs are underway to position PRRT in the therapeutic sequence of LC and will be key to confirm these results but also to properly assess short‐ and long‐term tolerance of this therapeutic strategy, allowing the determination of the best treatment line sequencing.^124^, ^125^

#### 2.6.3 Recommendations (Figure 3)

Based on available evidence, the panel recommends everolimus either as first‐line treatment in AC or as second‐line post‐SSA, in patients with progressive advanced LCs and ThC (**2b, B**). Although there is evidence for the efficacy of MKIs in non‐pretreated patients (i.e., axitinib, surufatinib), cabozantinib is the only currently approved angiogenesis inhibitor in western countries for the treatment of epNETs upon progression or, intolerance, to at least one prior systemic therapy other than SSA (**2b, B**). For patients with comorbidities that increase risk of everolimus‐related toxicity, cabozantinib may be considered upfront. When ORR is a treatment goal, PRRT in SRI‐positive disease (within clinical trials or off‐label if available) (**4, B**) or chemotherapy (temozolomide‐based—preferable in d‐MGMT—or oxaliplatin‐based in p‐MGMT or MGMT unknown) could be recommended as alternative or third‐line treatment option (**2b, B**). In the absence of better evidence, PRRT is generally recommended as fourth‐line salvage option as well as tumour agnostic‐based molecularly targeted therapies in eligible patients (**4, B**).

In patients with uncontrolled functioning tumours or increased 5‐HIAA levels above 3–5 UNR, anti‐secretory agents should be maintained and combination of systemic interventions with LRT (see Q8 and Q9) should always be discussed (**4, B**).

#### 2.6.4 Specific considerations regarding thymic carcinoids or MEN1 status

The strategy in MEN1 or ThC patients is similar as in sporadic LC. One large series of patients with advanced ThC has reported best ORR with chemotherapy, including cisplatin and carboplatin, and very few patients eligible for PRRT (5%).^26^

Q12: Is there a role for immunotherapy or agnostic molecular therapy in advanced progressive refractory lung and thymic carcinoids?

Although the preclinical and biological rationale is weak, several trials have tested immunotherapy in NETs.^126^, ^127^, ^128^, ^129^, ^130^, ^131^, ^132^ Overall, single immune checkpoint inhibition (ICI) has yielded negative results in molecularly unselected tumours, although some studies have suggested some efficacy in LC (i.e., spartalizumab reported an ORR of 17% for thoracic carcinoids versus 3% for GEP‐NETs), PDL1‐positive tumours (>10%), tumours with high tumour mutational burden (>10 mut/Mb) or *ARID1A* genomic mutations.^128^, ^129^, ^133^ No obvious superior results were observed with dual PD1‐CTLA4 checkpoint inhibition or with ICI combined with angiogenesis inhibitors.^130^, ^131^, ^134^, ^135^, ^136^, ^137^, ^138^, ^139^ Finally, combination of ICI with chemotherapy could be more promising but deserves additional trials.^132^ Delta‐like ligand 3 (DLL3) protein, involved in Notch signalling, is expressed in 30%–40% of LC and has emerged as a new and attractive tumour‐specific target.^140^ DLL3‐targeted strategies are being developed in small cell lung carcinoma (SCLC) and extrapulmonary NECs with very promising results.^141^ Tarlatamab, a bispecific T‐cell engager targeting DLL3, has been recently granted accelerated approval by the FDA for previously treated extensive‐stage SCLC. Efficacy of these strategies in LC, however, remains to be addressed.

A retrospective study that performed tumour NGS in 116 advanced NEN patients, including 45 LC/ThC primaries, showed that the mTOR pathway and *MEN1* alterations were the two most frequently found in more than 10% of patients.^142^ mTOR pathway alterations did not seem to predict everolimus efficacy. Among other targetable molecular alterations, anaplastic lymphoma kinase (ALK) rearrangements have been described in a few LCs and some case reports have documented responses to ALK inhibitors.^143^ Other druggable genomic alterations (i.e., *NTRK*, *RET* or *NRG1* fusions; *EGFR*, *BRAF* or *KRAS* mutations) have been rarely reported in LC.^144^, ^145^, ^146^

#### 2.6.5 Recommendations

Immunotherapy is not recommended outside of clinical trials except for pembrolizumab for the two FDA‐approved tumour‐agnostic indications, MSI (pembrolizumab, dostarlimab) or TMB‐H tumours (pembrolizumab).

Due to their scarcity, screening of ALK translocations or other targetable molecular alterations is not routinely recommended, but may benefit a few patients and therefore, tumour molecular profiling may be considered if available in patients with no other available treatment options (**5, D**).

#### 2.6.6 Specific considerations regarding thymic carcinoids or MEN1 status

Even if the biology of MEN1 and ThC differs from LC, it currently remains untargetable.

### 2.7 Follow up

Q13: What is the recommended follow‐up after surgery for localised lung carcinoid?

The risk of recurrence as defined by RFS or time to recurrence has been seldom studied in LC. Rate of recurrence of LC ranges between 6 and 28% after a median follow up of 42–87 months.^35^, ^147^, ^148^, ^149^, ^150^ Recurrences are local in 5%–16% of cases, local or regional in 16%–25% of cases or occur at distant sites in 55%–84% of cases.^58^, ^149^, ^150^, ^151^ Recurrences are more frequent in AC, ranging from 25% to 35%, as compared to the 3%–7% observed in TC after a median follow‐up of 51–91 months.^3^, ^58^, ^151^ Since the median follow‐up of all studies is below 10 years, the shape of the RFS curve after 10 years is unknown. The benefit of surveillance for early detection of recurrences on time for a potentially curative rescue therapy with achievement of a new R0 status is unknown. No single study has reported the modality and interval of imaging follow‐up.

It should be noted that in metastatic LC, conventional imaging as defined above remains the gold standard of follow‐up, associated with symptom assessment and a few biomarkers like 5‐HIAA and UFC.

#### 2.7.1 Recommendations (Figure 4)

**FIGURE 4:**
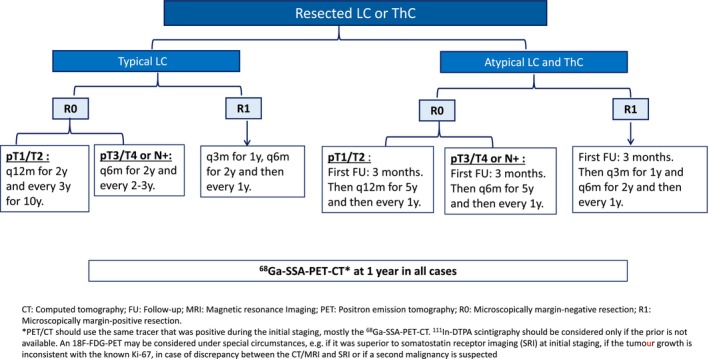
Follow‐up recommendations after surgery for lung (LC) or thymic (ThC) carcinoids.

Symptoms related to the tumour and to functioning syndrome should be regularly monitored; however, routine biomarker assessment is not recommended due to limited sensitivity and specificity. Bronchoscopy should be performed during follow‐up in the presence of symptoms, particularly in patients with unresected primary tumours, those who underwent sleeve resection, or in case of abnormal imaging if the resected tumour was visible by bronchoscopy at initial staging.

Given the need for long term follow‐up, conventional surveillance with multiphase CT scan is increasingly being replaced by lower‐radiation imaging modalities, such as MRI of the mediastinum and abdomen combined with low‐dose non‐contrast lung CT (**5, C**). Follow‐up should also include SRI‐PET, in addition to CT/MRI, 12 months following surgery and whenever equivocal findings are observed on conventional imaging or if there is clinical suspicion of relapse or high risk of recurrence. An ^18^F‐FDG‐PET‐CT may be considered if it demonstrates superior performance to SRI‐PET at initial staging.

For patients with R0‐resected TC, the first follow‐up is recommended at 12 months for pT1‐T2‐N0 tumours and at 6 months for pT3‐T4‐N0 tumours. In AC, a first imaging follow‐up is recommended at 3–6 months. Prolonged monitoring is advised, ideally within dedicated, registered prospective cohorts, although imaging intervals can be progressively extended (figure 4 as an exemple). Prospective validation with standardized imaging follow‐up is expected.

An echocardiography by a cardiologist experienced in CHD is recommended during the follow‐up only in cases of established CHD, the presence of CS and/or increased 5‐HIAA levels (**4, B**) (see Q9).

#### 2.7.2 Specific considerations regarding thymic carcinoids or MEN1 status

Follow‐up imaging of patients with MEN1 and their relatives who carry a MEN1 germline mutation should include specific follow‐up of the pancreas and the pituitary gland as per current guidelines.^152^

ThC have a higher risk of R1 resection and of progression or relapse and should be followed using the same recommendations as for AC (**5, C**).

## 3 CONCLUSIONS AND FUTURE PERSPECTIVES

This ENETS guidance paper provides up to date practical advice on the diagnosis and management of LC and ThC. However, several critical areas remain underexplored and warrant further research:

- TNM classification and long‐term outcomes: There is a need for prospective cohort studies in well characterised populations for WHO and TNM classifications at baseline, to support the development of a dedicated TNM classification for LC. Long‐term detailed follow‐up (beyond 10 years) is particularly relevant in these patients to properly assess disease‐free, disease‐specific, and OS, both for R0 and R1 resected tumours.
- Biomarkers and molecular characterisation: The clinical utility of biomarkers in LC remains unclear due to insufficient clinical annotations coupled to each sample but also large series of controls for specificity evaluation. Early CgA decline (1 month) may constitute a surrogate of outcome (as suggested in RADIANT‐2), but its value in LC remains unproven; further studies are needed to validate CgA and 5‐HIAA as response surrogates. Circulating tumour DNA or other liquid biopsy approaches may enable the molecular characterisation of advanced disease and may play an increasing role in monitoring tumour evolution and the tumour environment. Development of CHD in asymptomatic LC patients with increased 5‐HIAA levels requires prospective confirmation.
- Role of functional imaging: The impact of systematic use of SRI‐PET in the preoperative setting to improve staging accuracy, prognostic classification and follow‐up strategies deserves to be properly assessed. Emerging functional imaging modalities such as ^18^F‐FDG‐SSTR‐PET‐TC, ^68^Ga‐SSTR‐PET‐MRI or ^64^Cu‐SARTATE PET are under evaluation and may offer enhanced diagnostic performance in the near future. Their role as predictors of response to PRRT requires further investigation.
- Risk stratification and adjuvant therapy: Prospective registration in the adjuvant setting is highly encouraged, including well‐characterised patient populations in terms of WHO classification, TNM staging, Ki‐67, and surgical margin status (R status), as well as adjuvant therapy received, if any, and modality of follow up must also be described. Future research should prioritise the identification of predictive biomarkers for residual disease and RFS, and the standardisation of follow‐up modalities.
- Secretory Syndromes Clinical Impact and Management: The prevalence and clinical burden of secretory syndromes require further dedicated studies. Specifically, the role of locoregional and systemic therapies in their control, the influence of the primary tumour versus metastatic sites or other factors on CS severity and CHD development, and the identification of reliable biomarkers are key to optimise antisecretory strategies. Hormone‐related symptoms and 5‐HIAA levels should be analysed on a regular basis in clinical trials with dedicated endpoints.
- Therapeutic trials and biomarker‐driven research: A more refined prognostic risk‐stratification approach, along with a broader implementation of tumour growth assessment prior to intervention in asymptomatic patients, is expected to improve clinical decision‐making for patients with advanced LC/ThC. The development of phase II/III RCT specifically dedicated to LC with homogeneous inclusion criteria and integrated biomarker programmes is essential to strengthen the evidence base and guide therapeutic decision‐making. Long‐term safety profiles, predictors of toxicity and response to treatment, and evaluation of novel agents, including epigenetic therapies and next‐generation optimised radiopharmaceuticals, are active research areas of great interest. Research should be fostered by international networking and should consider an expected higher degree of uncertainty as recommended in the EU Rare Cancer Agenda 2030. Pharmaceutical company involvement to develop new drugs in rare tumours is urgent, as well as policymakers and regulatory health authorities recommendations regarding applicable methodology and criteria for drug development, approval and reimbursement in this context.

In conclusion, robust prospective data collection, incorporation of molecular diagnostics and the development of new therapeutic strategies are highly needed to improve management and outcomes of LC and ThC patients. Prospective real‐world cohort studies, including assessments of symptoms, biomarkers and quality of life, and inclusion of patients in ongoing trials must be prioritised to advance care and standardise future clinical practice. To this end, multidisciplinary and international collaborations are underway but they lack adequate political support.

## CONFLICT OF INTEREST STATEMENT

The authors declare no conflicts of interest.

## Supporting information


**Table S1.** Selected questions on the diagnosis, treatment and follow‐up of patients with lung and thymic carcinoids.
**Table S2.** Level of evidence and grade of recommendations.

## Data Availability

The data that support the findings of this study are available from the corresponding author upon reasonable request.
