# Supplementary material for: European Society of Neuroendocrine Tumors (ENETS) 2025 guidance paper for lung and thymic carcinoids
Source: J Neuroendocrinol. 2026 Apr 6;38(4):e70174. doi: 10.1111/jne.70174 (PMC13053111; doi:10.1111/jne.70174)
Supplement: Supplementary file 1 — Table S1. Selected questions on the diagnosis, treatment and follow‐up of patients with lung and thymic carcinoids. Table S2. Level of evidence and grade of recommendations. [file JNE-38-e70174-s001.docx]

**Table S1.** Selected questions on the diagnosis, treatment and follow-up of patients with lung and thymic carcinoids

| Q1 | Is there an added value of Ki67 or other pathological or molecular markers to the current WHO 2021 classification of lung and thymic carcinoids? |
| --- | --- |
| Q2 | What is the most appropriate imaging for accurate TNM staging and is there a need for a dedicated TNM classification? |
| Q3 | Which biomarkers and when should they be used in daily practice? |
| Q4 | When can observation or wedge/segmentectomy be considered a safe approach in localised lung carcinoid patients as compared to standard lobectomy plus lymph node dissection? |
| Q5 | Which is the best therapeutic strategy in case of R1 resection for a localised lung carcinoid? |
| Q6 | Role of locoregional treatment on primary tumour or metastatic sites? |
| Q7 | Is there a role for adjuvant therapy in M0R0 resected typical and atypical carcinoids? |
| Q8 | How should Cushing syndrome be managed in the preoperative or palliative settings in lung and thymic carcinoid patients? |
| Q9 | How should carcinoid syndrome be managed in the non-curative setting? |
| Q10 | What is the recommended first and second line therapy to control slowly progressive advanced typical and atypical carcinoids? |
| Q11 | What is the recommended first and second line therapy to control advanced atypical carcinoids or progressing typical carcinoids? |
| Q12 | Is there a role for immunotherapy or agnostic molecular therapy in advanced progressive refractory lung and thymic carcinoids? |
| Q13 | What is the recommended follow-up after surgery for localised lung carcinoid? |

**Table S2.** Level of evidence and grade of recommendations

| Level of evidence | Therapy | Diagnosis |
| --- | --- | --- |
| 1a | Systematic review (with homogeneity) of RCT | 1. An independent, masked comparison with reference standard among an appropriate population of consecutive patients |
| 1b | RCT (with narrow confidence intervals) |  |
| 2a | Systematic review of cohort studies | 2. An independent, masked comparison with reference standard among non-consecutive patients or confined to a narrow population of study patients |
| 2b | Individual cohort studies or low quality RCT |  |
| 3a | Systematic review (with homogeneity) of case-controlled studies | 3. An independent, masked comparison with an appropriate population of patients, but reference standard not applied to all study patients |
| 3b | Individual case-controlled studies |  |
| 4 | Case series (and poor-quality case-controlled studies) | 4. Reference standard not applied independently or masked |
| 5 | Expert opinion without explicit critical appraisal | 5. Expert opinion without explicit critical appraisal |
| Grade of recommendation | | |
| A | Strong | |
| B | Moderate | |
| C | Low | |
| D | Very low | |

*RCT: randomized controlled trial*
